# The complete mitochondrial genome of *Smaragdina nigrifrons* (Coleoptera: Eumolpidae): characterization and phylogenetic position

**DOI:** 10.1080/23802359.2019.1688710

**Published:** 2019-11-12

**Authors:** Wen-Jia Yang, Shu-Yan Yan, Dao-Chao Jin

**Affiliations:** a Provincial Key Laboratory for Agricultural Pest Management of Mountainous Regions, Institute of Entomology, Guizhou University, Guiyang, P. R. China;; b Guizhou Provincial Key Laboratory for Rare Animal and Economic Insect of the Mountainous Region, College of Biology and Environmental Engineering, Guiyang University, Guiyang, P. R. China

**Keywords:** *Smaragdina nigrifrons*, leaf beetle, mitogenome

## Abstract

The complete mitogenome of *Smaragdina nigrifrons* (GenBank accession number MN584924) is 15,903 bp in length, and includes 13 protein-coding genes (PCGs), 22 transfer RNA genes (tRNAs), 2 ribosomal RNA genes, and a control region. The overall base composition was as follows: A, 38.18%; T, 35.25%; C, 15.97%; and G, 10.60%, with a total of A + T content of 73.43%. Eleven reading frame overlaps and thirteen intergenic regions were found in the mitogenome of S. *nigrifrons*. All 13 PCGs are initiated with the typical ATN codons, and are terminated with either the complete TAA/TAG codons or a single T residue. All tRNAs possess the typical cloverleaf secondary structures except for *trnS_1_* (AGN). Phylogenetic analyses showed that *S*. *nigrifrons* was closely related to *Cucujus clavipes*, which was consistent with the conventional taxonomy.

The leaf beetle, *Smaragdina nigrifrons* (Coleoptera: Eumolpidae), is a major agricultural pest and distributed widely in China. The beetle cause severe economic damage to many plants, including jujube, corn, bean, abacus, and millet. The adults of *S*. *nigrifrons* feed on plant leaves and bite them into holes and notches (Gao et al. 2019). In this study, the specimen of *S*. *nigrifrons* were collected from Fanjing Mountain in Guizhou Province of China (N27°53′, E108°48′), and stored in 95% ethanol and registered in the Insect Specimen Room of Guiyang University with an accession number GYU-Col-2019001.

The complete mitogenome sequence of *S*. *nigrifrons* (GenBank accession number MN584924) is 15,903 bp in length, harboring the typical set of 37 mitochondrial genes which include 13 protein-coding genes (PCGs), 22 transfer RNA genes (tRNAs), 2 ribosomal RNA genes (*16S rRNA* and *12S rRNA*), and a control region (Boore 1999). All genes have the similar strands and location with that of click beetle, *Agriotes hirayamai* (Lin et al. 2018). The overall base composition of *S*. *nigrifrons* mitogenome was as follows: A, 38.18%; T, 35.25%; C, 15.97%; and G, 10.60%, with a total of A + T content of 73.43%. The AT-skew and GC-skew of this genome were 0.040 and −0.202, respectively. Amongst the 37genes, 14 genes (*trnQ*, *trnC*, *trnY*, *trnF*, *trnH*, *trnP*, *trnL_1_*, *trnV*, *nad1*, *nad4*, *nad4L*, *nad5*, *16S rRNA*, and *12S rRNA*) were located on the light strand (L-strand), while the remaining 23 genes were encoded on the heavy strand (H-strand).

The total length of 13 PCGs of *S*. *nigrifrons* was 11,034 bp. The 13 PCGs in the mitogenome initiated with the typical ATN as start codons (ATG for *cox2*, *cox3*, *atp6*, *atp8*, *nad4*, *nad5*, and *cob*; ATT for *nad3*, *nad4L*, *nad6*, and *cox1*; ATA for *nad1* and *nad2*). Ten PCGs used typical termination codons TAA and TAG in *S*. *nigrifrons*, while only three PCGs (*cox3*, *nad4*, and *nad5*) stop with the incomplete termination signal T. There were 11 overlap regions comprising a total length of 212 bp and the largest spacer (92 bp) resided between *trnA* and *trnN*, and 13 intergenic spacer regions ranging in length from 1 to 8 bp, comprising a total length of 42 bp. The length of the tRNAs varied between 62 bp (*trnE* and *trnT*) and 70 bp (*trnK*), comprising a total length of 1447 bp. All 22 tRNAs possessed the typical cloverleaf structure except for *trnS_1_* (AGN), which lacked the dihydrouridine arm and occurred commonly in most insects (Ohtsuki et al. 2002; Yuan et al. 2016). The *16S rRNA* and *12S rRNA* were 1283 and 740 bp in length, with the A + T contents of 77.71 and 73.51%, respectively. The control region was 1229 bp in length and had a remarkably high A + T content (81.86%), which was located between *12S rRNA* and *trnI*.

Phylogenetic analyses were performed with respect to the concatenated amino acid sequences of 13 PCGs. A neighbor-joining tree was constructed by the program MEGA version 7.0 (Kumar et al. 2016). The result showed that *S*. *nigrifrons* was closely related to *Cucujus clavipes* (Figure 1), which was consistent with the conventional taxonomy.

**Figure 1.**
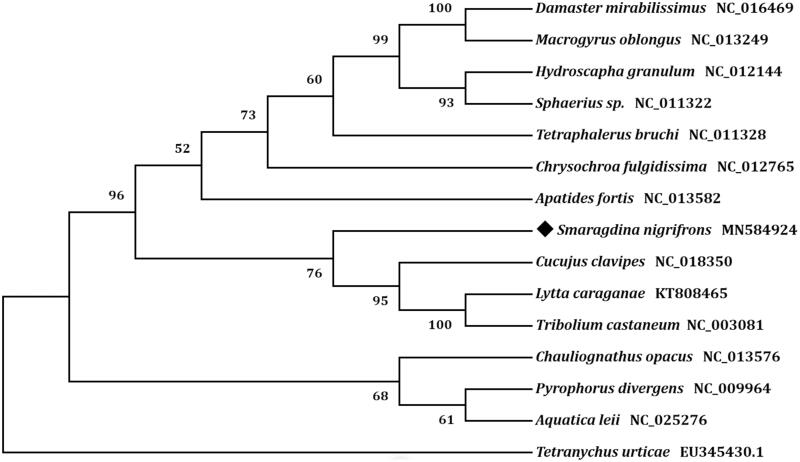
Phylogenetic tree showing the relationship between *Smaragdina nigrifrons* and 13 other beetles based on neighbor-joining method. Beetle determined in this study was labeled with black diamond. *Tetrancychus urticae* was used as an outgroup. GenBank accession numbers of each species were listed in the tree.
